# Caring for providers to improve patient experience (CPIPE): intervention development process

**DOI:** 10.1080/16549716.2022.2147289

**Published:** 2022-12-12

**Authors:** Patience A. Afulani, Edwina N. Oboke, Beryl A. Ogolla, Monica Getahun, Joyceline Kinyua, Iscar Oluoch, James Odour, Linnet Ongeri

**Affiliations:** a Epidemiology and Biostatistics Department, University of California, San Francisco (UCSF), San Francisco, CA, USA; b Institute for Global Health Sciences, University of California, San Francisco, San Francisco CA, USA; c Research Department, Global Programs for Research and Training, Nairobi, Kenya; d Center for Virus Research, Kenya Medical Research Institute, Nairobi, Kenya; e County Executive Committee, Migori, Kenya; f Migori County Referral Hospital, Migori, Kenya; g Center for Clinical Research, Kenya Medical Research Institute, Nairobi, Kenya

**Keywords:** Person-centered maternity care, stress, implicit bias, Kenya, respectful maternity care

## Abstract

A growing body of research has documented disrespectful, abusive, and neglectful treatment of women in facilities during childbirth, as well as the drivers of such mistreatment. Yet, little research exists on effective interventions to improve Person-Centred Maternal Care (PCMC)—care that is respectful and responsive to individual women's preferences, needs, and values. We sought to extend knowledge on interventions to improve PCMC, with a focus on two factors – provider stress and implicit bias – that are driving poor PCMC and contributing to disparities in PCMC. In this paper we describe the process towards the development of the intervention. The intervention design was an iterative process informed by existing literature, behaviour change theory, formative research, and continuous feedback in consultation with key stakeholders. The intervention strategies were informed by the Social Cognitive Theory, Trauma Informed System framework, and the Ecological Perspective. This process resulted in the *‘Caring for Providers to Improve Patient Experience (CPIPE)’* intervention, which has 5 components: provider training, peer support, mentorship, embedded champions, and leadership engagement. The training includes didactic and interactive content on PCMC, stress, burnout, dealing with difficult situations, and bias, with some content integrated into emergency obstetric and neonatal care (EmONC) simulations to enable providers apply concepts in the context of managing an emergency. The other components create an enabling environment for ongoing individual behavior and facility culture change. The pilot study is being implemented in Migori County, Kenya. The CPIPE intervention is an innovative theory and evidence-based intervention that addresses key drivers of poor PCMC and centers the unique needs of vulnerable women as well as that of providers. This intervention will advance the evidence base for interventions to improve PCMC and has great potential to improve equity in PCMC and maternal and neonatal health.

## Background

Person-centred maternal care (PCMC) has become a priority in the global discourse on quality of care due to the prevalence of disrespectful, abusive, and neglectful treatment of women during facility-based childbirth. PCMC emphasises that maternity care is respectful of and responsive to individual patient preferences, needs, and values [1]. PCMC has direct effects on maternal and neonatal outcomes through effective communication, patient engagement, supportive care, and improved psychosocial health [2–5]. It is also associated with better patient safety, trust, and higher patient and provider satisfaction [6,7]. Poor PCMC on the other hand leads to poor outcomes through lack of, delayed, inadequate, unnecessary, or harmful care [2]. In particular, poor PCMC deters women from giving birth in health facilities [8–11]. Further, PCMC is critical from a human rights perspective, as everyone has a right to be treated with dignity and respect during pregnancy and childbirth [12–15].

A growing body of research has identified that the barriers and facilitators of PCMC operate at multiple levels. At the individual and interpersonal levels, poor PCMC drivers include inadequate provider knowledge of PCMC, poor provider attitudes about PCMC, power asymmetry between patients and providers, as well as provider stress and burnout [16–20]. Providers often share that disrespect and abuse are either unintended or necessary to help women during childbirth [17,21,22]. Others blame women’s disobedience and lack of cooperation, and perceive abuse as an acceptable means of gaining compliance to ensure a good outcome [21,23]. Poor PCMC is also driven by systemic and social drivers such as lack of accountability mechanisms and broader social and gender norms that facilitate and normalise disrespect and abuse [16,17,20,21,24–26]. These systemic and social drivers are often difficult to influence; thus, many interventions focus on individual drivers such as provider knowledge of PCMC [27–29]. These systemic barriers however interact with individual drivers in complex ways to affect PCMC. An example is the role of provider stress, burnout, and difficult patient-provider interactions in PCMC [19,20,30,31].

Stress is the psychological and physiological response to environmental stressors [32]. The stressors providers in SSA encounter are numerous and include: feelings of inadequacy in the face of high mortality; an overwhelming workload from staff shortages; inability to provide best practice due to lack of drugs, supplies, and equipment; being required to manage complications beyond their competency; financial strain from poor remuneration; and poor working conditions with insufficient basic resources [33–35]. The influence of provider stress on poor PCMC cannot be overemphasised given the large body of evidence showing that prolonged stress without adequate coping mechanisms leads to burnout [36,37], which manifests as poor attitudes towards patients [38–40]. Stress is also a key factor in difficult situations, which are due to a combination of patient characteristics, caregiver characteristics and skills, and a stressful environment [41]. The labour and delivery ward in low-resource settings creates the perfect storm for these difficult situations – a combination of a difficult work environment, stressed and demotivated providers with rigid expectations of how a woman in labour should behave, and patients whose needs are not being met and are labelled as ‘difficult’ [19]. Providers’ ability and capacity to respond to patients in these difficult and stressful situations without resorting to disrespect and abuse, is thus of critical importance. Further, provider stress and burnout are associated with poor health outcomes such as depression, cardiovascular disease, and premature mortality, and is of great cost to the health system vis-a-vis absenteeism and high staff turnover [36,38,42]. Addressing provider stress and burnout is therefore of broader public health importance, beyond improving PCMC [36].

While PCMC is generally sub-optimal, it is often overlooked that PCMC is not equally experienced by all women: the most vulnerable groups tend to receive the poorest care [43–46]. These disparities in PCMC result in disparities in facility deliveries – with lower rates among women of low socioeconomic status (SES), adolescents, and minority groups, who are more likely to be mistreated in health facilities [43,44,47]. One important reason for differential PCMC is bias: the negative evaluation of one group relative to another. Bias can be explicit/conscious or implicit/unconscious [48]. Implicit bias operates at a sub-conscious level and does not require a person to endorse or devote attention to its expression. Instead, it can be activated quickly and unknowingly by situational cues such as a person’s skin colour, accent, clothes, or other outwardly appearance [48,49]. Implicit bias is widespread in every society, but the predominant types of biases differ across contexts [50,51]. In the US, where implicit bias has been extensively studied, anti-Black bias among physicians manifests as lower likelihood of evidence-based prescribing and lower quality interpersonal care for Black as compared to White patients [52–54]. Although implicit bias is not a prominent research topic on drivers of PCMC, it is recognised that the way people are treated in healthcare settings is a reflection of the broader societal norms and behaviours [34]. The mistreatment of women of lower SES reflects a society where differential treatment based on SES is normative [34,45]. Provider bias therefore reinforces the patterns of abuse [55,56], hence the need to address it in interventions to improve PCMC.

Despite the importance of PCMC, documentation of poor PCMC across different settings globally, and identification of its drivers, a significant gap still remains in evidence-based interventions to improve it in LMICs [27,57–59]. Most interventions in Africa focus on training providers on respectful maternity care [59–63]. Other interventions include improving the health facility environment, working with policymakers to encourage greater focus on disrespect and abuse of women, and strengthening linkages between the facility and community for accountability and governance [28,29,64]. Evidence on the effectiveness of these interventions is however limited, and there remains a dearth of interventions to improve PCMC that are grounded in theory [27,58,59]. There is also a critical need for interventions that address the fundamental factors driving poor PCMC such as provider stress and burnout. Further, interventions have not approached the problem with an equity lens, hence do not emphasise the experiences of poor and other marginalised groups. No published intervention on PCMC in SSA has explicitly addressed PCMC, provider stress, and implicit bias in an integrated fashion. We believe addressing these key drivers of poor PCMC is critical to improving PCMC and reducing inequities.

The goal of this project was to develop a theory and evidence-based intervention to improve PCMC that addresses provider stress and implicit bias among maternal health providers in LMICs. We hypothesised that a multicomponent intervention that enables providers to identify and manage their stress and to be conscious of their biases and how their actions affect marginalised women’s experiences will improve PCMC and reduce disparities in PCMC. The specific aims were to (1) Design the intervention through an iterative process in consultation with key stakeholders in the planned intervention region; (2) Pilot the intervention to assess its feasibility and acceptability; and (3) Assess preliminary effect of the intervention. In this paper we focus on the intervention design process (aim 1). The results of the other aims will be described in future manuscripts.

## Methods

### Participants and setting

The target population for this project is health care workers in maternity units as well as other support units in Migori County, Kenya. This site has previously been described [9,65,66]. To summarise: Migori County, located in western Kenya, has eight sub-counties. The county population is approximately one million, with an estimated 40,000 births annually. The estimated maternal mortality ratio is high at 673 deaths per 100,000 live births compared to 495/100,000 nationally. There is one county referral hospital, seven sub-county hospitals, and several health centres, dispensaries, and faith-based and private health facilities. The health care worker patient ratios are 32 nurses, 19 clinical officers, and 4 doctors per 100,000 people. Based on the most recent Kenya demographic and health survey, 53% of births in the county occurred in health facilities, compared to the national average of 61%.

### Needs assessment: formative research phase 1 and 2

The need for this intervention was informed by findings of our initial research in Migori, which focused on understanding the extent of PCMC as well as the barriers and facilitators to providing it. Thus, this served as the initial formative research for the intervention design. Data collection for this initial period starting in 2016 included surveys and in-depth interviews with over 1000 women who had recently given birth and 49 providers in the county. This was followed by a second phase of data collection in 2019 with about 100 providers focused on better understanding the role of provider stress and bias in PCMC. The methods and findings from this initial formative work in Migori County are published, including quantitative and qualitative interviews with women highlighting gaps in PCMC [9,44,65,66], as well as qualitative and quantitative interviews with providers to understand drivers of poor PCMC [19,20,25], and to assess extent of provider stress and bias [30,31,67]. We summarise the relevant findings from phase 2 to provide context for the intervention components.

First, the work highlighted that provider stress, burnout, and bias as well as difficult situations during childbirth were key drivers of poor PCMC [19,20,25,66]. Other provider-level drivers included inadequate knowledge and skill on various aspects of PCMC, perceived lack of time, forgetfulness, self-protection, and assumptions about women’s knowledge and expectations [19,20,25,66]. Further, using an adapted Implicit Association Test (IAT) and situationally specific vignettes to assess implicit and explicit SES biases, respectively, we showed that providers had implicit biases in their associations between difficult patient characteristics and patient SES attributes. Providers also had explicit biases and incorrect assumptions about poorer women and care expectations (e.g. poor women did not expect providers to introduce themselves to them and would not sue if something went wrong, or that once a woman is present at a facility, she has consented to all subsequent care). Data highlighted that differential treatment was linked to women’s appearances, assumptions about who is more likely to understand or be cooperative, women’s ability to advocate for themselves or hold providers accountable, ability to pay for services in a timely manner, and situational factors related to provider stress and burnout [67]. These factors interacted in complex ways to produce PCMC disparities. Many of the drivers of poor PCMC and sources of bias are modifiable and could be addressed in trainings; yet, only 22% of providers surveyed in our study sites in Migori County reported participating in a training to improve patient-provider interactions [67].

Further, 96% of providers surveyed were experiencing moderate to high stress, while 85% were experiencing low to high burnout [31]. When asked what causes them the most stress at work, most providers (55%) stated high workload [68]. Lack of supplies or equipment was the second most common leading cause of stress (15%), followed by poor salaries (8%). Other sources of stress were frequent staff turnover, personal/family problems, incompetence of other providers, attitudes of superiors, colleagues, and patients, and death of a patient. Within the last year, 34%, 37%, and 55% of providers reported they had been treated in a way that was disrespectful or humiliating by their superiors, colleagues, and patients respectively. Close to half of providers (42%) reported they had ever lost a baby or mother during pregnancy or childbirth; with 57% reporting this had happened in the last year. Seven per cent had thoughts of suicide. Yet, few providers have had training on how to cope or deal with stress – over 80% of the providers had never received stress management training – although almost all (98%) reported they would like such a training. Similarly, 88% reported that they had no readily available access to psychological and emotional support, from a counsellor, psychologist, psychiatrist, or other mental health professional, although almost all (94%) reported they would like to have access to such support. Eighty-four per cent (84%) had no access to workplace peer support, although 92% wanted such support. Thirty-six per cent had no mentor in the country, although all wanted to have a mentor in the county. These findings underscored the need for an integrated multi-level intervention that addressed several of the drivers of poor PCMC, as well as supported providers.

### Intervention development

**Figure 1.**
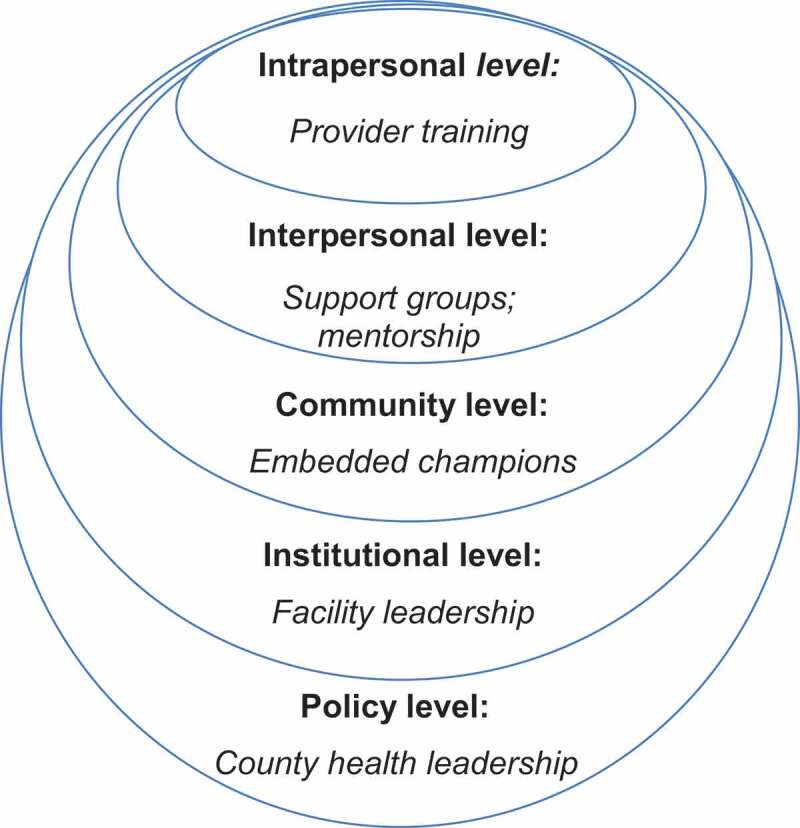
Ecological approach.

**Figure 2.**
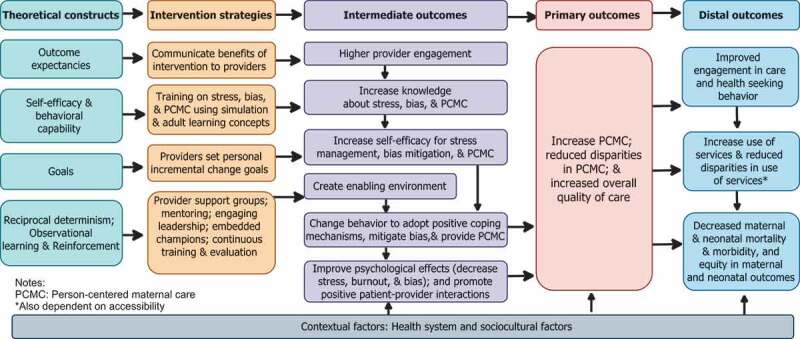
Conceptual framework.

Drawing on the existing literature on interventions to improve PCMC [27,57], reduce stress and burnout [69–71], and mitigate the effects of bias [72–74], we first designed the CPIPE intervention to target key drivers of poor PCMC using social and behavioural science theories – Ecological Perspective, Social Cognitive Theory (SCT), and Trauma Informed System (TIS) frameworks–to inform the intervention strategies. The ecological perspective recognises that behaviour is influenced at multiple levels and underscores the importance of enabling environments for individual behaviour change [75]. We therefore identified intervention components at different levels of influence (Figure 1), to create an enabling environment for individual behaviour change efforts. SCT describes a dynamic process in which personal factors, environmental factors, and human behaviour exert influence upon each other (reciprocal determinism). SCT posits that if individuals have a sense of self-efficacy, they can change their behaviour even when faced with obstacles. Also, people are more likely to change if they believe the activity has benefits to them (outcome expectancy); and if there are tangible goals, positive role models (observational learning), and reinforcement [76]. These SCT constructs informed the intervention strategies (Figure 2). For example, the intervention will include a training component, which emphasises benefits of stress reduction and coping strategies for the provider, as well as for women and their babies, to decrease resistance and increase their engagement. The training will also use simulation, a technique of choice in training professional teams, to evoke real-life scenarios in an interactive fashion to increase self-efficacy [77], and adult learning techniques such as self-directed inquiry [78]. Finally, the TIS framework recognises stress as a source of trauma to the system, which if not addressed leads to numbing, reactivity, and depersonalisation [79,80]. TIS supports ‘reflection in place of reaction, curiosity in lieu of numbing, self-care instead of self-sacrifice, and collective impact rather than siloed structures’ [80]. This philosophy informed the overall intervention approach.

For the training content, we proposed that the CPIPE intervention includes both didactic and interactive sessions addressing key drivers of poor PCMC. Given that the sources of stress are difficult to avoid in the life of a provider in a low resource setting, our approach focuses on the factors that influence the stress response – which is providers’ perceptions of the stressors, coping strategies, and their general wellbeing [32]. For implicit bias, a first step towards mitigation is recognising and creating structures to minimise it [50]. Additionally, people have to be concerned about the effects of bias to be motivated to identify and learn to replace biased responses with responses more consistent with their goals [72]. Further, existing psychological theories posit that people are inherently complex, with multiple and often contradictory patterns of selves. Thus, it is possible to reduce the effects of people’s bias through activities that elevate the alternative selves and goals that people endorse, without actually removing their deep-seated biases – referred to as sidelining implicit bias [81,82]. We therefore utilised content from our prior research to increase bias awareness and mitigation, as well as an overall focus on the caring provider whose goal is to provide PCMC – with particular attention for the needs of the most vulnerable whom they may unintentionally mistreat.

To create an opportunity for providers to practice the training content in the context of providing clinical care, we planned to integrate the content on PCMC, stress, bias, and difficult situations into an emergency obstetric and neonatal care (EmONC) simulation and training developed by PRONTO International. The PRONTO training kit, called a PRONTOPack™, includes a hybrid birth simulator called PartoPants™ (a modified pair of surgical scrubs with all anatomical landmarks necessary for delivery) worn by a patient actress, which creates a highly realistic experience that centres the patient [83]. During each simulation, one provider plays the role of the patient, enabling them to engage with the patient experience. This also creates the opportunity to practice providing PCMC while managing an obstetric emergency. Simulations are followed by a debrief, which provides the opportunity for discussion and feedback about the patient and provider experience [83]. This drew on our prior work in Ghana where we successfully integrated PCMC into PRONTO’s simulation and training curriculum and showed that this was acceptable to providers and was an effective means to improving PCMC in SSA [84,85].

For the mentorship, peer support, embedded champions, and leadership engagement components, we decided to collect additional data in phase 3 (described below) to assess the relevance, feasibility, and acceptability of these components and to tailor them to the context. In addition, we sought feedback on the training content and approach as well as its composition, duration, and location.

### Acceptability of intervention plan: formative research phase 3

This phase of research involved in-depth interviews with 21 stakeholders in the county, including nurses, midwives, clinical officers, support staff, community members, and county/sub county health management teams. The participant list was collaboratively developed in consultation with the study team, who had prior experience and in-depth knowledge of the context in which this intervention will be implemented. The interviews were conducted remotely over phone from January to March 2021 due to restrictions during the early phase of the pandemic. Semi-structured guides were used by two interviewers, who were core members of the study in the previous phase, to explore perceptions regarding the intervention strategies, as well as preferences for structure, content, format, modality, frequency, location, etc. Question formats included describing the planned intervention to respondents and asking for their perceptions and concerns about it. We also asked open-ended questions about their preferences for how the various components should be implemented. Interviews were audio-recorded transcribed and coded by the study team utilising a collaborative deductive coding framework. Codes were then queried, and excerpts analysed using a thematic approach. Additionally, at the beginning of this phase of the work, we established a local advisory board to advise on the project to ensure the intervention was relevant and feasible to the context and to develop local ownership – a critical component to the sustainability of the project. We summarise the results of this research which informed the final intervention plan below, organised by the five intervention components. Representative quotes are presented in Table 1.

**Table 1.** Representative quotes for themes from formative research on intervention plan.

Theme	Subtheme	Representative quotes
*Training topic*	***PCMC***	‘In the human approach, I think in certain areas where we have also worked with this strategy it has worked well because it allows the client to express themselves. Here is where we find clients will tell you the truth because we want them to be involved in certain things on them, like respectful maternity care, we all expect women to deliver just in one position, but when you get to talk to the mother as an individual, actually when we discuss with other mothers, then they tell us that probably that may not be the best position for them to deliver.’ **Nurse 23**
	***Implicit bias***	‘ … a training with such kind of content, will of cause bring in consciousness of the health care worker so that our patient is treated with dignity for purposes of improving the quality of care. So also, from my personal experience, this kind of topics are not in the conventional curriculum and that is why am saying that this is a very good selection in the area so that the general training will help bridge the gap that is existing.’ **County official 01**
	***Stress***	‘So the contents, I think being that these are the areas that you are going to train the staffs on, I think they are quite in order and the one I like is the one on stress and coping mechanisms because most of us health care workers we have our different stress and our workplace do not help us to cope with it and so we may not even be able to cope with our stresses, so I think it is a very good topic for us, as health care workers,working in the maternity to attain.’ **County official 02**
	***Difficult situations***	‘ … it is a nice one because sometimes you have a difficult situation and you don’t know what to do next, it is like you are hitting a dead end … so if providers get that information, then it will help then to think out of the box.’ **Nurse 08**
*Training Format*	***Mixing cadres***	‘ … when we are combined in that training, then the support staff will appreciate the wellbeing of the client at the same time the clinical staffs will appreciate, and when they are brought together and the share the experience then at the end of the training the teamwork be evident, and we will learn as health care provider that we can work without the support staff and equally the support staff. I think they should be brought together.’ **Nurse 17**
	***PRONTO Integration***	‘It will really help because you know when you do PRONTO, this is something that you are seeing being done in the simulations and when you see something, it sticks more, you can’t forget it, so I think it’s a good idea.’ **Nurse 11**
	***Duration***	… Normally trainings of two to three days, I find with the limited number of staffs that we have, taking someone out from work for one whole week is not workable. If it is a five days or six days training, then you can take two days train this week and the complete the trainings the next week again three days. We break them into two days per week to make it not take the providers away for too long.” **County official 02**
	***Location***	‘I think a mix of both hospital and outside facility is important as you can mentor someone outside the facility, maybe if the facility is your cause of stress, then there is an aspect of it that you may feel that there is not much change done to it so I think a mixture of both in the facility and one or two sessions away from the facility normally works very well.’ **County official 03**
*Peer support*	***Value of peer support***	‘Peer support that you come together as people of the same working area, you talk to each other like organizing one day a week and share what you have undergone both bad and good, difficult, and encouraging situations, you help each other and share. So, it may be [that] you meet and discuss and develop some action plan on moving to the next step.’ **Nurse 20**
	***Mixing cadres***	‘I still feel that we should group them according to cadres, for example a support may fear to say some points because she feels inferior to the doctors so when they are put in their cadres then they can freely speak of what is affecting them’ **Nurse 19**
	***Number of people in a group***	‘Too many people will not be productive, because in peer counseling I think people want to share and I think when everybody wants to share, and the group is too large people will lose concentration.’ **Nurse 04**
	***Meeting frequency***	‘It depends because people need to be supported so I don’t know how the program can do that but, if possible, we can have it monthly, but if it turns to be hard on the program then we can have it after every three months.’ **Clinical officer 15**
	***Meeting format***	‘ … so, in the group you will say you are sharing confidentiality but somebody somewhere who is not good at maintaining the confidentiality might leak the information… Remember we are dealing with stress and the best way is to talk with the affected person face to face.’ **Nurse 08**
*Mentorship*	***Value of mentorship***	‘It is good, and it improves output … In our medical set up we lack mentorship because seniors are left on their own and the juniors too are on their own so you have to grow as you are moving and there is nothing else…Number two it goes beyond mentorship and counseling in stress management the mentors do, so to me mentorship is very important especially if it done by those with experience on work and not just level of education or your boss at place of work.’ **Facility leader 01**
	***Choosing mentors***	‘.that is good but again it has some limitation when it is done in the same facility, for example when you pair me with my senior here to mentor it would be so well at time but then bad at the same time and will not be successful because in the process of carrying our daily activities in the health facility, you cannot avoid rubbing shoulders with your senior or junior. If it has to be a senior, then it has to be from a different facility within the country someone you have not rub shoulders.’ **Nurse 17**
*Embedded champions*	***Value of embedded champions***	‘That approach is good because it has been tried in some other lines and we have seen it working,we have a number of projects going on in this facility and those champions have ensured that the staffs are equal to their tasks, and it has helped to improve the indicators to a greater extent and so I think that it is also important to have such people because when we sleep then they are the people who help to wake us up.’ **Nurse 25**
	***Selecting champions***	‘We should see people who are dedicated because we know dedicated people in an institute, we can go through matron. We don’t just choose someone who is only after money. we need a dedicated person who even during training shows a lot of interest and dedication.’ **Support staff 005**
	***Keeping champions engaged***	‘I think once you train [champions], what you may also do is to support them in meetings… Then maybe the other way that we could also approach this or make use of a mechanism that has been working for this referral hospital is that they have CMES meetings, once we have trained the champions, some of the topics that they can use to present to the staffs on CMES they give talks on stress management and other soft skills is important because in CMEs we discuss technical things like … new-born or maternity issues but very few of them look at the softer skills so through the CMEs the champions trained will use it to cascade it to other providers’. **County official 03**
*Leadership engagement*	***Value of leadership engagement***	‘I think the approach is a good one, you know when you include the leadership, and they get to know some of the challenges and issues that health care workers have in the health care delivery system. This is because part of their role is actually to allocate resources in the health care system and if they know some of the challenge.’ **Community health volunteer 09**
	***Initial engagement***	‘ … there should be an inception meeting with the sub-county team because they are the once who will participate even in electing the participant who needs to attend the training, so they need to be aware to plan.’ **Nurse 05**
	***Maintaining engagement***	‘You can take advantage of the monthly meetings, the study team and technical team can take advantage of the meetings as they are assured meetings and by doing that you will be able to know and give a report of what happened last month, this month and how are we today as opposed to only waiting until something happens. At facility level you can safely engage the existing structure.’ **Facility leader 01**

#### Training

In general, respondents approved of all the training topics proposed to them and many noted how a training on the topic would be helpful to them. For instance, for PCMC almost all respondents highly recommended the training as something that would improve their performance at work. One respondent however noted some providers might be reluctant to disclose their mistreatment of patients in a training due to fear of reprimand (e.g. job termination). Thus, it was important to provide a safe space where participants can talk about their experiences without fear of judgement or punishment. For implicit bias, most respondents affirmed the presence of bias in the way providers treat patients and felt that the training was relevant. It was, however, acknowledged that although implicit bias is prevalent and the training needed, how the content is delivered might affect how participants receive it. Similarly for stress and coping mechanisms, all respondents acknowledged that providers in the maternity unit undergo stress due to several reasons, and providers will appreciate a training on dealing with stress and will participate effectively. Most respondents held a favourable view of integrating the new content with the PRONTO simulation training, having participated in prior PRONTO trainings. However, a couple of respondents raised concerns about feasibility of integration, specifically related to time constraints and the size of PRONTO’s package – noting that a smaller package could be more effective.

Regarding the composition of people at each training, the views were mixed. Some thought the mixing of cadres was a good idea because it reflected their working conditions and provided an opportunity to foster teambuilding skills and promote teamwork. Others, however, thought the mixing of cadres was not a good idea because of different levels of training, knowledge, and understanding between providers and support staff. Others noted prejudice and the feeling of inferiority in junior staff and support staff would discourage them from fully engaging in the training. When asked how long a reasonable period was to take providers out of work for training, the average suggested duration of training was between 2–4 days (ranging from one day to two weeks). Some respondents recommended that the training be held off-site (i.e. hotel or conference centre) to give them a break from the usual work stressors and limit distractions, while others preferred facility-based training due to convenience and increased access to the trainings. Suggested approaches to enhance engagement included making the trainings interactive, such as using scenarios, simulations, dramatisation, and team-building activities. Additionally, they recommended carving out time for participants to have tea together to discuss and ‘share and build each other up.’ Leadership/management involvement and support as well as follow-up trainings were also considered facilitators of engagement.

#### Peer support

Most respondents greatly appreciated the strategy of peer support groups. They noted that although there were no such groups for health care providers, it has worked well for people living with HIV and for family planning programmes. Respondents cited that in these groups’ providers can debrief after having difficult work experiences. It was also noted that peer support groups will enable providers to bring up issues they all experience, listen to each other and freely discuss how they are dealing with the issues raised among themselves, without supervisors. Like the training, views regarding composition of peer support groups were mixed: Some respondents thought mixing different cadres of providers will create the opportunity to discuss shared experiences. Most however preferred cadre-specific groups – noting that some providers (such as support staff) might not feel comfortable enough to openly share their experiences in the presence of other cadres. Most of the respondents preferred in-person peer support group meetings (as opposed to an online platform) where providers can meet each other, express themselves easily, and have assurance of confidentiality. Meeting frequencies mentioned ranged from weekly to quarterly. Using WhatsApp groups for peer support was acceptable to a few, but others noted they were already on several WhatsApp groups, and discussions on those groups are not confidential. It was also noted that some of the providers do not have access to smart phones.

#### Mentorship

All the respondents appreciated the need for a mentorship programme, as this will bring together providers for knowledge exchange. Respondents recommended that the programme comes up with a key area on what the providers need to be mentored on based on gaps we have identified. It was noted that there are some existing mentorship programmes within the county (focused mostly on clinical topics) hence the need to come up with different focus areas for this programme to avoid duplication. Respondents however had mixed reactions about how mentors and mentees should be paired. Some respondents liked the idea of paring senior and junior staff as mentors and mentees respectively. But others noted that using years of experience may not be effective, given many of the leaders in top positions in the health department are junior in terms of years of experience, but are more qualified. Some also suggested that junior staff may be able to mentor senior staff in areas they have expertise in, as senior staff can have limited knowledge on some topics. It was thus recommended that, what needs to be considered is expertise and competence, and not just seniority.

#### Embedded champions

All respondents acknowledged that using embedded champions is good for sustainability. Some respondents recommended that the best way to go about selecting the champions is to have two per facility, so that when one is transferred, the other person can continue to train the rest. Some respondents noted that champions should be people who add value to the project; and are vocal, passionate, and dedicated. Some recommended we identify the most active person during training in a facility as the embedded champion, while others noted all provider cadres should be considered to avoid biases and wrangles in the facilities. Suggested ways to motivate embedded champions included having periodic meetings, supportive supervision by the project team, providing some form of motivation, having continuous assessment and designated roles for the champions, involving facility leaders, and forming WhatsApp groups for champions. One respondent suggested that the champions should be invited for existing continuous medical education (CME) meetings in the referral hospital to learn both soft and technical skills.

#### Leadership engagement

This was noted as critical for ownership and sustainability. Thus, involving the county leaders in the health system and forming a community advisory board (CAB) to represent all stakeholders, including at the community level, was laudable. Some respondents noted that many projects tend to work with county coordinators and side-line the facility in charge. It was thus commendable that the project involved the facility in charge, as this was important. It was also noted that involving both county and facility leadership in the project would enable them to get to know the challenges that providers encounter. Suggested ways to maintain leadership engagement included inviting leaders to the trainings, identifying a focal person for the project at the facility level to facilitate project activities, and having periodic meetings with the leaders to update them and discuss planned project activities. It was also suggested that the project team connects with county officials to discuss how the project activities can be integrated into the annual county work plan.

#### Final intervention approach

Following analysis of the formative research data, we presented the findings to the CAB and made several decisions regarding the final intervention plan and approach to implementation. This included an agreement to proceed with all the intervention components as proposed (Table 2), with careful attention to how they were presented. For example, we focused on creating a safe space where providers can be comfortable speaking up and engaging without fear of reprisal. We also continued with the plan to integrate the proposed topics into the EmONC simulation training, but reduced the number of simulations to only two during the initial training, which will also include didactic and interactive sessions on topics listed in Table 2 in the form of case-based learning, teamwork activities, hands-on practice, and reflective sessions. While acknowledging the concerns of those who thought it might not a good idea to combine different cadres of staff, we decided to go ahead and combine them for the training since most approved of this, and this was a way to improve teamwork and communication and promote interprofessional development. We also decided to limit the initial training days to two days, followed by monthly refreshers over the 6-month intervention period. All maternity providers in the intervention facilities will be invited to participate in the training to facilitate a person-centred facility culture change. To help manage patient needs and keep number of participants to a reasonable level, given protocols for the COVID-19 pandemic, facility leads will divide providers in their facility into two groups who will attend alternate 2-day training sessions. The initial training was to be held offsite, while the monthly refreshers will be held onsite in each facility on days and times identified as appropriate at the facility (e.g. during designated CME times). The initial training days and times were selected in consultation with the CAB and county and facility leadership, who were all invited to participate in the training. Other recommendations such as pre and post assessments for training, providing pamphlets, interactive activities, tea breaks, etc., were incorporated into the training plan.

**Table 2.** CPIPE intervention strategies.

**(1) Training**
Person-centred maternity care Understanding stress and burnout and developing positive coping mechanisms. Introduction to mindfulness Bias awareness and mitigation Dealing with difficult situations Emergency obstetric and neonatal care Teamwork and communication Mentorship and peer support
**(2) Peer support groups**
Groups for providers to meet with other providers of their cadre, and discuss issues they are facing, brainstorm solutions, and provide support to one another.
**(3) Mentorship**
Mentor-mentee relationships that provide the opportunity to coach junior providers on professional development, work-life balance, clinical skills, career advancement and other topics. Mentors develop their mentorship and leadership skills.
**(4) Embedded champions**
To facilitate ongoing engagement and sustainability at the facility level, we identified facility champions who lead in organizing and facilitating peer support groups and refreshers at their facilities and serve as role models
**(5) Leadership engagement**
Engagement of County leadership at the onset of the project through a community advisory board, regular updates of the study and findings, and discussing systemic gaps that impact provider stress and bias.

For peer support, we agreed these will comprise groups of people of similar cadres (nurses/midwives, support staff, doctors/clinical officers) within each facility. A leader will be selected from each group within the facilities to coordinate and lead in-person meetings of the group each month. WhatsApp will only be used for sharing resources. Based on discussions about mentorship during the formative research, we fielded a short survey to all providers in the intervention facilities to better understand their mentorship needs. The survey also included a list of people who were willing to serve as mentors, and potential mentees were asked to identify those they would like to have as mentors (to ensure mentees were paired with mentors they were comfortable with). Provision was also made for peer mentorship. For embedded champions, each facility will nominate two embedded champions following the initial training, who will serve as the central persons to facilitate monthly refreshers and coordinate peer support activities in their facilities. For leadership engagement, leadership at all levels will be included in the CAB, and CAB meetings will be organised quarterly for continuous leadership engagement. Per the earlier findings on gaps in individual psychological support, we reached out to inquire about the presence of clinical psychologists or mental health counsellors in the county who could provide individual counselling to providers and identified two. The two psychologists agreed to better meet the counselling needs of providers and shared their phone numbers to be given to intervention participants who could reach out to them as needed. The intervention is ongoing in the county. In subsequent manuscripts, we will discuss the implementation of the intervention, lessons learnt, participants perceptions of the intervention, and preliminary effectiveness on various outcomes.

## Discussion

*CPIPE* is a multicomponent theory and evidence-based intervention to improve PCMC that helps providers to identify and manage their stress and biases to prevent burnout and mitigate the effects of bias, while providing high-quality clinical care. The intervention was developed based on prior research, theory, and formative research, and has five components: training, peer support, mentorship, embedded champions, and leadership engagement. This research is important and innovative in several ways.

First, while it is recognised that providers in SSA work under very stressful conditions, provider stress and burnout is hardly considered in interventions to improve quality of care [34,86,87]. Our focus on stress management is particularly relevant and urgently needed given the impact of the COVID-19 pandemic on healthcare worker stress and burnout [88–92]. Interventions are also yet to address the role of difficult situations during pregnancy and childbirth. Second, while implicit bias, is a prominent topic in developed settings like the US [48,53,93], it is hardly acknowledged in SSA, especially in the context of clinical improvement interventions. Further, most interventions have not addressed the inequities in PCMC, and the impact of provider behaviour on the most vulnerable women who often receive the worst care. Thus, there is a need to develop and test rigorous PCMC interventions that consider the unique needs of vulnerable women. The use of the terminology of implicit bias in this intervention was intentional, as people are more open to participating when their actions are recognised as unintended. This approach is also based on our observations that exceptionalism in PCMC is usually positive – with some groups receiving better care than the norm – rather than negative, where the norm is good care and one group is singled out for poor treatment [44]. Put differently, providers are not intentionally mistreating poor women, rather the default is poor care and encounters with higher status groups cause providers to be intentional in their care, leading to better PCMC in these groups. The training approach therefore aims to increase intentionality during every patient encounter.

Addressing stress and bias together is significant, given research suggests that deeply held biases are more likely to emerge when people are stressed [49]. In addition, interventions such as mindfulness practices have been shown to have an impact on implicit bias through indirect effects on stress reduction [94]. Furthermore, addressing provider stress and implicit bias together acknowledges both the vulnerability and power of providers, and highlights outcomes not just for the patient, but also for the provider. As a result, providers are less likely to feel blamed and more likely to support such an intervention. The framing of the intervention ensures that providers receive support to cope with stressors that impact their ability to provide PCMC and mitigate the effects of bias.

The CPIPE intervention also leverages best practices for effective in-service training, including interactive techniques such as simulations, case-based learning, teamwork activities, hands-on practice, and immediate feedback on performance [95,96]. Further, the intervention addresses the interconnectedness of clinical care and PCMC using highly realistic simulation training. Quality of care includes both technical and interpersonal dimensions, thus the need for evidence-based interventions that address both [97]; however, most efforts to date have focused mostly on the technical dimensions. Additionally, the limited interventions to address PCMC are not delivered in the context of care provision during birth, where much of the abuse occurs. Instead, the existing interventions are delivered as stand-alone trainings on respectful maternity care [28,29,98]. Yet, disrespectful care emerges in the process of providing clinical care and is often triggered by stressful events like an obstetric emergency [18,19,99]. Highly realistic simulation training provides a unique opportunity to practice PCMC in a context, that mimics the stressful emergencies that can trigger disrespectful care. This study will bridge the gap in evidence-based approaches that concurrently enhance clinical and interpersonal skills of providers.

Most interventions to improve PCMC are not theory-informed [27,57]. Our intervention is innovative in the use of formative research and social and behavioural science theories to develop a multi-component multilevel intervention to address key drivers of poor PCMC. Training is a required step to initiate behaviour change but requires an enabling environment. Although peer support, mentorship, and use of embedded champions are recognised approaches to facilitate behaviour change, they have been underutilised in existing PCMC interventions. The intervention components of peer support, mentorship, embedded champions, and leadership engagement will create and sustain the enabling environment required for behaviour change.

Finally, we demonstrate a process of sustained engagement in a particular setting to ensure interventions address issues relevant and acceptable to the context. Often researchers go into a setting to fit in an intervention designed without the involvement of potential participants and with no previous engagement in the setting. CPIPE is a culmination of over 6 years of engagement with Migori County.

## Limitations and strengths

A limitation of this intervention is that it does not directly address structural factors such as shortage of health workers and essential supplies that affect provider stress and PCMC. These are important, but beyond the scope of an individual intervention. This intervention is to help providers cope and provide better care amid the structural deficiencies. It will complement efforts by government and other organisations to address the structural issues. Additionally, the strategies learnt from the intervention will help address issues they are dealing with, and the project provides several opportunities for providers to engage together and with leadership to discuss issues they are dealing with and brainstorm solutions. Furthermore, reducing burnout has the potential to reduce shortages from burnout-related absenteeism. There are currently no evidence-based interventions to improve PCMC in SSA that integrate content on health provider stress, burnout, explicit and implicit bias, as well as dealing with difficult situations within the context of strengthening emergency obstetric and newborn care. CPIPE seeks to bridge this gap by addressing these interrelated but under explored factors contributing to poor PCMC and to disparities in PCMC.

## Conclusion

Drawing on the existing research, social and behavioural science theories, and formative research in the intervention context, we designed the CPIPE intervention to target key drivers of poor PCMC. CPIPE addresses key factors rarely considered in MCH quality improvement efforts in SSA and bridges disciplines that have traditionally worked in silos. It is also timely, given the increasing evidence of poor PCMC in SSA, and the dearth of evidence-based interventions to improve it. In this paper we described the background, theory, and formative research leading to the development of the CPIPE intervention. In subsequent manuscripts, we will present data from a mixed-methods evaluation of the intervention describing the implementation process, lessons learnt during implementation, participants experiences and perceptions of the intervention, and preliminary effect of the pilot intervention on various outcomes. The CPIPE intervention will be among the first, if not the first, rigorously designed and evaluated intervention to improve PCMC that address key drivers of poor PCMC such as difficult situations, provider stress and burnout, and provider bias in an integrated fashion. We anticipate that the data from this pilot will be used to further refine the intervention and position it for a rigorous multisite intervention and evaluation, with a larger sample and longer follow up, to assess the effect of the intervention on provider and patient experience. This research will address a major gap in the efforts to improve PCMC and quality of care, with potential to impact MCH outcomes.

## Paper context

Theory and evidence-based interventions that address key drivers of poor person-centred maternal care and centre the unique needs of vulnerable women and providers are lacking. We used an iterative process informed by existing literature, behaviour change theory, and formative research to develop an intervention that bridges this gap. Implementation and evaluation of this intervention will advance the evidence base for interventions to improve person-centred maternal care and achieve equity in maternal health.
